# Navigating ethical challenges in the FORTEe randomised controlled trial: a multi-centre staff survey on exercise intervention for children and adolescents undergoing cancer treatment

**DOI:** 10.1186/s12910-026-01414-6

**Published:** 2026-02-25

**Authors:** Francesca Alt, Elias Dreismickenbecker, Francesca Lanfranconi, Adriana Balduzzi, Eila Watson, Hayley Marriott, Joachim Wiskemann, Nikolai Bauer, Rodolf Mongondry, Martin Kaj Fridh, Alejandro Lucia, Carmen Fiuza-Luces, Ronja Beller, Filippo Spreafico, Barbara Konda, Milica Stefanović, Heidi Diel, Mareike Kühn, Lena Wypyrsczyk, Norbert W. Paul, Marie A. Neu, Jörg Faber

**Affiliations:** 1 University Medical Center of the Johannes Gutenberg-University Mainz, Childhood Cancer Center Mainz, Mainz, Germany; 2 University Medical Center of the Johannes Gutenberg-University Mainz, Institute for the History, Philosophy and Ethics of Medicine, Mainz, Germany; 3 Fondazione Monza E Brianza Per Il Bambino E La Sua Mamma, Centro Maria Letizia Verga, Monza, Italy; 4 Pediatric Department, Fondazione IRCCS San Gerardo dei Tintori and School of Medicine and Surgery, Milano-Bicocca University, Monza, Italy; 5 Oxford Institute Applied Health Research, Oxford Brookes University, Oxford, UK; 6 School of Sport, Nutrition and Allied Health Professions, Oxford Brookes University, Oxford, UK; 7 Working Group Exercise Oncology, Department of Medical Oncology, Heidelberg University Hospital, Medical Faculty Heidelberg, Heidelberg University, National Center for Tumor Diseases Heidelberg, a partnership between DKFZ and Heidelberg University Hospital, Heidelberg, Germany; 8 Centre de Lutte Contre Le Cancer Léon Bérard, Lyon, France; 9 Department of Pediatrics and Adolescent Medicine, Copenhagen University Hospital, Rigshospitalet, Copenhagen, Denmark; 10 Department of Sports Sciences, Faculty of Medicine, Health and Sports, Universidad Europea de Madrid, Madrid, Spain; 11 Research Institute of the Hospital, 12 de Octubre (‘imas12’), Madrid, Spain; 12 University Hospital Essen, West German Cancer Center, Essen, Germany; 13 University Hospital Essen, Clinic for Pediatrics III, Hematology and Oncology, Essen, Germany; 14 Oncology Unit, IRCCS Istituto Giannina Gaslini, Genoa, Italy; 15 Forma 3D Ltd, Ljubljana, Slovenia; 16 University Medical Center Ljubljana, Ljubljana, Slovenia; 17 Fondazione IRCCS Istituto Nazionale dei Tumori, Pediatric Oncology Unit, Milan, Italy

**Keywords:** Childhood Cancer, Exercise intervention, Randomised controlled trial, Supportive Care, Child autonomy, Informed consent in paediatrics, Vulnerability in research, Moral distress, Burden-benefit assessment

## Abstract

**Background:**

The FORTEe randomised controlled trial (NCT05289739) investigates an exercise intervention for children and adolescents undergoing cancer treatment. Conducting research with this vulnerable population poses unique ethical challenges, including participant burden, child autonomy, and parental decision-making. This study explored the ethical experiences of healthcare professionals (HCPs) involved in the trial.

**Methods:**

A multicentre survey was conducted across ten clinical sites in Europe using a structured, browser-based questionnaire comprising both closed and open-ended questions. Domains included burden and benefits assessment, informed consent, child autonomy, parental influence and moral distress. Quantitative responses were analysed descriptively, while qualitative data underwent content analysis.

**Results:**

Seventy-nine HCPs participated, including exercise professionals (*n* = 30), physicians (*n *= 19), nurses (*n* = 8), psychologists (*n* = 5), social workers (*n* = 3), one social scientist, one medical ethicist and 12 individuals in other roles. A large majority of respondents (86.1%) agreed or strongly agreed that the overall burden-benefit balance of trial participation was appropriate, while 11.4% were unsure and 2.5% disagreed. Open-text responses described perceived challenges related to questionnaire burden, logistical demands, and emotional discomfort associated with control-group allocation. Informed consent procedures were generally perceived as appropriate. However, some respondents reported situations in which parental influence appeared to outweigh children’s expressed preferences, and difficulties were noted in assessing children’s evolving decision-making capacity. Among HCPs who described prognosis-related events (*n* = 45), 68.9% described experiences they associated with moral distress, particularly in relation to communication and decisions regarding continuation of participation.

**Conclusion:**

The trial's ethical climate was largely perceived as positive, though emotional and logistical burdens were noted. Reports of emotional discomfort and moral distress among staff highlight the ethical tensions between research integrity and individual well-being. Furthermore, divergent views on children’s capacity to give consent suggest the need for clearer guidance on paediatric autonomy and shared decision-making.

**Implications:**

Ethically sound paediatric research must address real-world burdens and emotional dynamics beyond procedural compliance. Findings from the FORTEe trial staff survey highlight the importance of flexible, child-centred approaches, sustainable access to beneficial interventions, and institutional structures that promote ethical reflection.

**Trial registration:**

Registered on ClinicalTrials.gov (NCT05289739, 21 March 2022) and in the German Clinical Trials Register (DRKS00027978, 28 January 2022).

**Supplementary Information:**

The online version contains supplementary material available at 10.1186/s12910-026-01414-6.

## Introduction

Clinical research in paediatric oncology involves unique ethical challenges, as investigators must protect vulnerable patients while advancing therapies [1]. Children with cancer not only face life-threatening illness, but also the long-term consequences of intensive treatments, ranging from cardiopulmonary dysfunction to profound psychosocial burdens [2]. These challenges highlight the need for integrated supportive care.

Exercise therapy has been shown to improve both physical recovery and psychosocial well-being in this population [3, 4]. “FORTEe-Get strong to fight childhood cancer “ trial investigates such interventions, which may enhance recovery beyond conventional treatment. While the therapeutic benefits of exercise are well documented [5], the ethical dimensions associated with implementing them in paediatric randomised controlled trials (RCTs) remain underexplored.

RCTs involving children must navigate unique challenges related to informed consent, autonomy and child protection [6, 7], while balancing scientific rigour with respect for young participants´ decision-making. Ethical guidelines emphasise securing both parental consent and child assent which require careful assessment of a child's capacity to comprehend the research aims and implications [8]. In exercise-based therapy, these challenges may be further nuanced. Exercise is often perceived as a safe and beneficial component of supportive care rather than a “research procedure”, potentially leading to misconceptions about the purpose, the likelihood of benefit and participation [9]. Additionally, exercise protocols consisting of multiple sessions may demand considerable physical effort from children undergoing intensive cancer treatment [10] underscoring the need for clear communication about the time and physical commitments [11]. Because exercise is often perceived as low-risk and beneficial within supportive care, the ethical justification of randomisation and temporary non-access to the intervention requires explicit consideration. In paediatric exercise-oncology trials, equipoise may be experienced differently by families and HCPs when an intervention is viewed as inherently helpful rather than experimental. The use of control conditions, including delayed or no access to exercise, can therefore generate ethical tension related to fairness, expectations of benefit, and post-trial access, particularly in the context of serious illness.

Pragmatic RCTs, such as the FORTEe trial, which test effects in real-world settings, bring additional complexities. As these trials integrate into clinical care, distinctions between research and standard care must be clearly defined alongside robust ethical oversight [12]. Recent evidence underscores that supportive care interventions, including exercise, mitigate treatment-related fatigue and improve health-related quality of life [13], highlighting the importance of rehabilitation and tailored training strategies that address both physical and psychosocial recovery [14, 15].

As the field evolves, it is essential to refine our approaches to paediatric cancer care, ensuring that therapeutic advancements are accompanied by a focus on the holistic needs of young patients. These ethical concerns are compounded by the emotional and moral challenges experienced by healthcare professionals (HCPs) involved in paediatric oncology trials. HCPs often face moral distress when navigating difficult decisions about trial participation, particularly when balancing the scientific goals of the trial with the potential harm or burden to participants. For example, the inclusion of control groups, the decision to discontinue trials after relapse, and concerns regarding post-trial access to beneficial interventions can contribute to significant emotional strain. These dilemmas highlight the importance of providing robust institutional support and creating ethical frameworks that prioritise the welfare of both participants and HCPs. Given the heightened vulnerability of paediatric cancer patients, adherence to ethical principles, such as beneficence, non-maleficence, autonomy, and justice, is essential to uphold research integrity and participant welfare [6].

Against this background, the present study reports a quantitative and qualitative survey of HCPs involved in the FORTEe trial. The survey explores ethical challenges encountered in practice, with particular attention to child assent and parental influence, perceived burden, fairness of control-group allocation, sustainability of access to exercise, and moral distress. By centring HCPs’ perspectives, the study aims to clarify how ethical principles are negotiated in real-world paediatric exercise-oncology research.

## Methods

### Informed consent and data protection

Before starting the survey, participants were informed about the survey aims, terms of use, data protection and the voluntary nature of participation. They were also explicitly informed that proceeding with the questionnaire constituted informed consent. The introductory screen further explained that the survey focused on HCPs ethical experiences related to their involvement in the FORTEe trial and that participants could skip any question. Demographic data (profession, gender, age range) and further questionnaire answers were collected, processed and stored using a pseudonym (Study ID) in accordance with the EU General Data Protection Regulation (GDPR) and local legislation.

### Research approach and Ethical approval

This mixed-methods staff ethics substudy was embedded within the randomised controlled, multi-centre FORTEe trial (NCT05289739) [16], which investigated an exercise intervention for children, adolescents and young adults (aged 4- 21) undergoing cancer treatment. The present manuscript reports a cross-sectional, browser-based survey of HCPs conducted between 2nd September and 11th October 2024 at ten FORTEe recruitment centres in Germany, Italy, Spain, France, Denmark, Slovenia and the United Kingdom. The survey was disseminated to HCPs involved in the FORTEe trial across participating sites, including physicians, nurses, psycho‐oncologists and exercise professionals. Participants’ involvement in the trial varied, ranging from direct delivery of the exercise interventions and patient care to indirect roles such as clinical oversight or coordination. Including professionals with differing levels of trial involvement allowed for the capture of a broad range of perspectives, including those not directly responsible for applying detailed protocol or eligibility criteria. This substudy surveyed HCPs only; children and parents participating in the FORTEe trial were not surveyed in this study.

The FORTEe trial was prospectively registered in the German Clinical Trials Register (DRKS00027978) on 28 January 2022 and on ClinicalTrials.gov (NCT05289739) on 21 March 2022. Ethical approval was first granted by the Rhineland-Palatinate Chamber of Physicians (lead committee; application no. 2021–15904) on 4 August 2021, with subsequent approvals obtained from local committees at each recruitment centre in accordance with national and local requirements.

To support understanding of the ethical context in which the trial was conducted, key procedural safeguards are briefly outlined here. Given the vulnerability of the study population, the FORTEe trial was conducted in accordance with Good Clinical Practice and the Declaration of Helsinki. Before enrolment, age-appropriate informed consent or assent was obtained and documented. Because minors cannot provide legally binding consent, written consent was obtained from a parent or guardian, while children were involved in discussions whenever appropriate. A two-step consent approach, including the use of age-appropriate materials and a minimum reflection period of 24 h, was implemented to minimise coercion. The protocol further stipulated that exercise sessions were to be paused or discontinued immediately if a child expressed resistance or showed signs of distress, regardless of parental consent. Detailed eligibility criteria are provided in Supplementary Material 1.

All trial personnel received standardised training delivered by the coordinating centre, covering Good Clinical Practice and age-appropriate communication techniques for consent and assent. Training also addressed recognition of medical or psychological contraindications and available support mechanisms for ethical or safety concerns. Ongoing refresher sessions, site-specific webinars, and site visits ensured consistent implementation across centres.

In this paper, *moral distress* refers to the psychological and emotional discomfort experienced when professionals feel constrained from acting in ways they believe to be ethically appropriate, consistent with established definitions in paediatric oncology research [17]. While this definition guided the analytic framework, the survey intentionally relied on participants’ self-reported interpretations of morally distressing experiences. As a result, responses reflect how HCPs themselves used and understood the term ‘moral distress’ in practice, rather than a strictly delimited theoretical construct.

### Recruitment and survey participants

The survey was distributed across all ten FORTEe centres and circulated by the designated site contacts to reach as many involved professionals as possible. To reflect the realities of multidisciplinary care, survey participation was not restricted to core FORTEe research staff but included wider clinical personnel such as nurses, psycho-oncologists, and other allied professionals. Involving these groups aimed to provide a more representative account of the ethical, practical, and clinical challenges of implementing the intervention in routine care.

### Data collection

The survey was conceptualised and developed by the multi-professional FORTEe ethics working group, with the aim of capturing HCPs perspectives on ethical aspects, perceived responsibilities, and role-specific challenges encountered during exercise interventions in paediatric oncology. The survey was developed around a set of predefined domains, which informed both the structure of the questionnaire and the subsequent analysis. An overview of the survey domains and their corresponding survey items is provided in Supplementary Material 3*.* These domains included: familiarity with inclusion and exclusion criteria; informed consent and parent–child decision-making dynamics; ethical issues during exercise testing and training; perceived burden-benefit balance; sustainability of the intervention and post-trial access; fairness in group allocation; and experiences of moral distress.

### Survey instrument

The survey questionnaire combined single-choice items, multiple-choice Likert scale items and open-text items, enabling both quantitative description and qualitative exploration of ethical experiences. The questionnaire was piloted with four HCPs from different disciplines to assess clarity, relevance, and interpretability of items; minor refinements in wording were made based on their feedback.

Participants could skip any question. Conditional follow-up questions were displayed depending on prior responses; consequently, not all items were viewed by all participants. Items not viewed reflect the survey’s skip-logic rather than active non-response. The full English version of the questionnaire is available as Supplementary Material 2. Survey items were organised into predefined descriptive domains; a detailed mapping of domains to survey items is provided in Supplementary Material 3.

The survey was administered electronically in English via a secure browser-based platform across all participating sites and disseminated through project-specific and institutional mailing lists. Questionnaire Data were collected via a web-based interface using the open-source NUM COMPASS web application [18]. The application was deployed on institutional infrastructure at the local study site. Each participant accessed the survey through a unique, non-personalised QR code; 175 QR codes were distributed. Participating sites were located in Germany, Italy, Spain, France, Denmark, Slovenia and the United Kingdom, where the primary local languages include German, Italian, Spanish, French, Danish, Slovene and English.

### Data analysis

Quantitative data were analysed descriptively using frequencies and percentages, calculated based on the number of valid responses per item. Missing responses were excluded on an item-by-item basis.

Qualitative free-text responses were analysed using a framework-guided descriptive approach [19, 20]. The predefined domains that structured the survey served as the initial deductive framework. Qualitative coding was conducted manually by the investigators using Microsoft Excel 2016 to organise responses within the predefined domains and to support inductive identification of recurring concerns and experiences. Free-text responses were independently coded by two investigators (F.A. and M.N.) with discrepancies discussed and resolved through reflexive dialogue.

For each domain, narrative summaries were developed and representative quotations selected to illustrate key findings. Overlapping issues across domains were subsequently grouped into higher-order, cross-domain themes. The predefined survey domains that structured data collection and analysis are summarised in Supplementary Material 3. These themes informed the integrative interpretation presented in the Discussion. Integration occurred at the interpretive level by relating quantitative patterns to qualitative findings within shared domains, rather than through formal statistical integration. This analytic approach enabled structured exploration of predefined domains while allowing for the identification of emergent patterns across participants’ narratives.

## Results

A total of 79 professionals participated in the survey, corresponding to a response rate of 45% (79 of 175 HCPs who were invited via distributed QR codes). Response rates were high for core demographic and consent-related items (up to 97.5%), but lower for ethically nuanced or open-ended items (59.5% to 97.5%).

### Cross-domain thematic synthesis

The integrative analysis of the eight a priori domains produced seven overarching themes, each reflecting recurrent concerns raised by HCPs: (1) ethical complexity in trial decision-making, (2) respect for child autonomy and valid assent, (3) burden-benefit balance and participant distress, (4) long-term sustainability of intervention benefits, (5) fairness in control-group allocation, (6) moral distress among staff in response to patient outcomes, and (7) perceived moral distress in patients and families.

These themes reflect patterns across survey domains and informed the organisation of the Results section.

### Respondent demographics

Seventy-nine professionals (*n* = 79) completed the survey, representing a range of clinical, research, and therapeutic backgrounds. Of the 78 respondents reporting gender, 57 (73.1%) identified as female and 21 (26.9%) as male. Professions represented included 30 exercise professionals (38.0%), 19 physicians/oncologists (24.1%), 8 nurses (10.1%), 5 psychologists (6.3%), 3 social workers (3.8%), with one social scientist (1.3%) and one bioethicist (1.3%). Twelve participants (15.2%) reported interdisciplinary roles and were coded as “other” (e.g. physiotherapy, neuropsychomotor therapy, clinical research coordination, education, osteopathy, and sports therapy). Most were aged 26–35 years (*n* = 46, 58.2%), followed by 36–50 years (*n* = 15, 20.3%), 9 (11.4%) aged 50 + years, and 8 (10.1%) aged 18–25. A summary of participants’ demographic and professional characteristics is presented in Table 1.

**Table 1:** Respondent demographics (*n* = 79)

Characteristic	Category	n (%)
Survey participation	Completed survey	79 (100%)
Professional background	Physicians/oncologists	19 (24.1%)
	Nurses	8 (10.1%)
	Social workers	3 (3.8%)
	Psychologist	5 (6.3%)
	Exercise professionals	30 (38.0%)
	Social scientist	1 (1.3%)
	Bioethicist	1 (1.3%)
	Other/interdisciplinary roles*	12 (15.2%)
Gender	Female	57 (73.1%)
	Male	21 (26.9%)
Age group (years)	18–25	8 (10.1%)
	26–35	46 (58.2%)
	36–50	15 (20.3%)
	≥ 50	9 (11.4%)

^*^Includes interdisciplinary roles such as physiotherapy, neuropsychomotor therapy, clinical research coordination, education, osteopathy, and sports therapy

### Ethical awareness related to inclusion and exclusion criteria

Of the 79 respondents, 64 (81%) reported familiarity with the FORTEe trial’s inclusion and exclusion criteria. Among the 67 who evaluated their adequacy, 59 (88.1%) considered them ethically appropriate, while 8 (12%) reported concerns regarding clarity. Qualitative comments (*n* = 10) referred to barriers such as insufficient training, unclear role expectations, and insufficient information.

### Knowledge of and experiences with informed consent

Sixty-six (83%) reported familiarity with the FORTEe informed consent process, while 13 (17%) did not. Of the 50 respondents who completed the follow-up items on experiential aspects of the informed consent process, 39 (78%) perceived little or no burden for patients or families. Five respondents (10%) reported burden on a single occasion, five (10%) reported recurrent burden, and one respondent was unsure (2%). Narrative responses described challenges related to parental influence on children’s willingness to participate, information overload and communication challenges related to cognitive or linguistic factors.

### Parent–child decision-making dynamics

This item was intended to capture ethically relevant tensions in shared decision-making about trial participation (e.g. assent, refusal, or continuation), rather than decision-making about clinical treatment.

Of 75 respondents, 14 (18.7%) reported encountering situations they considered ethically relevant regarding the relationship between patients and their parents in the context of trial participation. Two respondents (2.7%) reported such situations once and 12 (16.0%) on multiple occasions. Nine respondents (12.0%) were unsure, and 52 (69.3%) reported no such experiences.

Narrative responses illustrated situations in which parents encouraged participation despite initial reluctance expressed by the child, as well as situations in which children wished to participate but parental concerns led to refusal of consent. Respondents also noted instances of divergent views or communication difficulties between children and caregivers. One respondent stated:

*“[There were] noticeable differences of opinion between patients and their parents.”*

### Ethical events during exercise testing and training

Of the 77 respondents who answered this item, 62 (80.5%) reported no ethically relevant events during exercise testing or training. Ten respondents (13.0%) reported having witnessed at least one such situation, while five (6.5%) were unsure.

Written accounts (*n* = 5; 6.5%) commonly described difficulties interpreting children’s willingness to participate during physical assessments, particularly in younger children with fluctuating behaviour or motivation. In some cases, testing was discontinued due to uncertainty regarding assent. One exercise professional reported:

*“It was difficult to evaluate to which degree a young kid (6 years) wanted or did not want to be tested. He kept switching mood.., thus the testing was stopped.”*

### Perceived burden during trial participation

This section reports HCPs’ reports of perceived burden and their ratings of the burden-benefit balance of trial participation, focusing on burden experienced by children and families during the FORTEe trial, as perceived and reported by staff. For the purposes of analysis, perceived burden was defined a priori as any instance in which trial participation was perceived by HCPs to cause distress, compromise autonomy or privacy, or interfere with wellbeing.

Seventy-seven respondents commented on perceived burden among trial participants or their families. Of these, 51 (66.2%) reported observing no burden, three (3.9%) reported burden on a single occasion, 18 (23.4%) reported burden on multiple occasions, and five (6.5%) were unsure.

Open-text responses identified three reported sources of perceived burden. First, questionnaire volume and frequency were described as burdensome, particularly in the context of intensive treatment schedules. Respondents reported fatigue or emotional strain associated with repeated or lengthy questionnaires. One respondent noted:

*“Several families told me that the amount of questionnaires was too big and too frequent.”*

Second, sensitive or intrusive questionnaire content was described as distressing for some families, particularly questions addressing personal or socio-economic circumstances during periods of heightened emotional vulnerability:

*“We had a few patients who did not appreciate the socio-economic questions because we referred to sensitive family situations.”*

Third, respondents reported difficulties related to children’s comprehension of questionnaire items and the timing of assessments, particularly during intensive treatment phases:

*“Few parents reported that the child was uncomfortable in answering certain questions. Other parents complained that the child could not understand the meaning of the question.”*

In a subsequent item assessing the perceived balance between burden and benefit, all 79 respondents provided a rating. Twenty-six respondents (32.9%) strongly agreed and 42 (53.2%) agreed that the overall burden-benefit balance of trial participation was appropriate (86.1% combined); nine (11.4%) were unsure and two (2.5%) disagreed.

### Long-term sustainability of the exercise intervention

This section reports HCPs’ assessments of post-trial exercise provision following completion of the FORTEe intervention.

Of the 63 respondents, 21 (33.3%) strongly agreed and 33 (49.2%) agreed that participants’ exercise needs were adequately met after completing the trial- a combined 82.5% (*n* = 52) expressing a positive view.

Qualitative responses indicated that structured exercise programmes were available beyond the trial period at their sites. These were described as having been integrated into routine care and, in some cases, extended beyond former trial participants. Respondents most frequently referred to the presence of local infrastructure, such as on-site exercise facilities, trained staff, or video-based exercise options. At some sites, this infrastructure was described as having been established or strengthened during the FORTEe trial. One respondent stated:

*"We have implemented [exercise] therapy in standard care … even after participation in the study has ended."*

In contrast, sustainability concerns were reported at sites with fewer resources. Eight respondents (12.7%) disagreed and three (4.8%) strongly disagreed that post-trial exercise needs were adequately met. Reported barriers included limited staffing, lack of institutional embedding, and the absence of exercise programmes outside the trial context. One respondent noted:

*“Outside of the FORTEe project there is no standard exercise programme for children or families within our hospital or community setting.”*

In addition, 25 respondents (31.7%) reported difficulties explaining the discontinuation or absence of post-trial exercise opportunities. Among these, 12 respondents (15.2%) indicated that such situations occurred frequently or occasionally.

### Fairness for control group participants

Among respondents, 26 (32.9%) reported no difficulties related to control group allocation, while 28 (35.4%) reported encountering ethically or practically challenging situations on multiple occasions and nine (11.4%) reported such situations once. A further 16 respondents (20.3%) were unsure or did not provide a clear response.

In open-text responses, respondents described emotional reactions among some children and families following allocation to the control group. These accounts most commonly referred to disappointment or frustration, particularly among children who were highly motivated to participate or who associated exercise with perceived benefit. In some cases, respondents reported reduced engagement with trial procedures or decisions not to participate. One respondent noted:

*“Some children were sad to be in the control group or decided not to participate in the study because they didn't want to be in the control group.“*

Difficulties were most often described in relation to families whose children had positive experiences during the intervention phase or were allocated to the control group:

*“It is sometimes hard to tell motivated patients that they are part of the control group, but it helps to tell them that they have the chance to take part after the intervention phase.”*

Respondents also described situations in which intervention and control group participants were present in shared spaces. In these contexts, some respondents reported difficulties adhering to trial procedures while providing care to children allocated to the control group. As one respondent stated:

*“I find it ethically difficult to [exercise] with children if the person in the same room was assigned to the control group and [was] therefore not allowed to take part in [exercise] therapy.”*

A small number of respondents explicitly articulated normative ethical concerns regarding the withholding of exercise from control group participants:

*“It is difficult not to train someone who needs it.”*

*“I personally believe that not training a patient is unethical.”*

Additional logistical challenges were reported at centres with limited spatial separation between groups. In response, respondents described strategies such as offering alternative activities, emphasising voluntariness, or maintaining contact with control group participants to reduce feelings of exclusion.

### Experience of moral distress

The following findings distinguish between moral distress experienced by HCPs themselves and distress perceived in patients or families, as reported from the HCP perspective. Although moral distress was analytically defined as a professional experience, respondents frequently used the term broadly to describe emotionally and ethically challenging situations encountered in the context of serious illness, including distress arising from close relationships with families or from witnessing patient suffering.

Of the 77 respondents, 52 (67.5%) reported witnessing cancer progression, relapse, or death among FORTEe trial participants on multiple occasions, while four (5.2%) reported such experiences once. Eight respondents (10.4%) had not encountered these situations, and 13 (16.9%) were unsure.

### Moral distress among staff in response to prognosis-related events

Fifty-one respondents (64.5%) completed the follow-up question asking whether the prognosis-related events were associated with moral distress. Twenty respondents (39.2%) did not report any associated moral distress. Among the remaining respondents, 12 (23.5%) reported moral distress affecting HCPs themselves, 10 (19.6%) observed distress primarily among patients or families, and nine (17.6%) reported distress affecting both staff and families.

All respondents who completed this item were subsequently invited to elaborate in an open-text field. A total of 45 respondents provided narrative comments. Of these, 31 (68.9% of those providing narrative detail) described experiences they explicitly associated with moral distress in the context of disease progression, relapse, or death. Twelve responses (26.7%) referred to other context-specific concerns not clearly framed as moral distress. Among the 31 respondents who described moral distress, 21 provided sufficiently detailed and concrete accounts of situations they personally experienced as morally distressing. These responses were categorised into five non-mutually exclusive categories (Table 2). The most frequently reported category was emotional distress related to patient relapse or death (*n* = 8; 38.1%), followed by distress arising from emotionally significant relationships with families (*n* = 6; 28.6%), and ethical unease regarding the continuation of trial participation in terminal situations (*n* = 5; 23.8%). Three respondents (14.3%) referred to strain related to witnessing parental suffering, and three (14.3%) reported stress associated with research-related demands during periods of clinical decline. Percentages exceed 100% because multiple categories could be reported. These descriptions illustrate how clinicians operationalised the concept of moral distress in their responses, often encompassing situations of emotional, relational, and ethical strain that extend beyond narrowly defined constraint-based accounts. One respondent reflected:

*“When a child who had previously participated in the [FORTEe] trial relapsed, it was difficult to explain to the family that they would not re-enrol and complete the FORTEe intervention a second time. This was particularly difficult when the child had enjoyed the sessions and found them to be a helpful coping strategy during treatment. While the child and family understood, it was hard to see them disappointed.”*

**Table 2:** Categories of HCP reported moral distress

Category of moral distress	Description	Frequency (n)	%
Patient relapse or death	Distress related to witnessing relapse or death among enrolled children	8	38.1%
Emotional bonds with families	Distress linked to close emotional relationships with patients/families	6	28.6%
Balancing research with patient well-being	Ethical unease regarding trial participation decisions	5	23.8%
Parental and family distress	Emotional strain related to witnessing patients or parental suffering	3	14.3%
Research-related demands during decline	Stress associated with research procedures or trial requirements during period of clinical deterioration	3	14.3%

Categories of moral distress reported by HCPs (*n* = 21 valid respondents; multiple responses allowed). Percentages sum to more than 100% because respondents could report more than one category

### Perceived distress among patients and families (HCP perspective)

A separate survey domain assessed HCPs´perceptions of distress among patients and families during trial participation.

Twenty-six respondents (33%) reported perceiving distress among patients and families during trial participation. Reported sources frequently related to child suffering (*n* = 10; 38.5%), and clinical deterioration during the trial (*n* = 3; 16.7%). Additional concerns included communication difficulties (*n* = 4; 15.4%), uncertainty regarding trial procedures (*n* = 3; 11.5%), and emotional pressure associated with decision-making under stressful circumstances (*n* = 3; 11.5%). Distress during discussions about trial continuation in the context of disease progression was noted by five participants (19.2%), and psychological burden related to reconciling hope with medical reality was also reported by five respondents (26.9%).

Eighteen respondents (22.8%) provided narrative accounts, of which 15 were thematically coded. As multiple themes could be identified per respondent, cumulative percentages exceed 100%. These accounts do not represent direct statements from patients or families but reflect how HCPs´ interpretations of distress experienced by families.

Across these narratives, perceived family distress was most commonly described in situations of severe clinical decline, including bereavement following a child´s death, relapse leading to exclusion from continued participation, and unexpected clinical deterioration during the trial. Respondents also highlighted emotionally demanding situations in which families struggled to reconcile hope associated with the exercise intervention with worsening prognosis. In addition, practical constraints—such as isolation protocols or bone marrow transplantation—were described as exacerbating family strain by limiting participation in exercise sessions. One respondent noted:

*“They were upset about the prognosis of the disease.”*

## Discussion

This study explored HCPs´ ethical challenges experienced during their involvement in a multi-centre paediatric exercise-oncology RCT. While the overall ethical climate of the FORTEe trial was perceived as positive, respondents reported recurrent tensions related to informed consent and child assent, perceived burden, fairness of randomisation, post-trial sustainability, and moral distress. Taken together, these findings illustrate how ethical issues were experienced and managed by HCPs in everyday clinical-research practice when working with a highly vulnerable population, and how ethical challenges extend beyond formal protocol adherence.

### Child assent, parental influence, and respect for emerging autonomy

A central ethical issue identified in this study concerned the interpretation of children’s assent and the role of parental influence in trial participation. Although formal consent procedures were in place, respondents described situations in which children’s willingness to participate was difficult to assess, particularly when emotional states fluctuated or reluctance was expressed non-verbally. These challenges were reported most often in the context of diagnosis, intensive treatment phases, or disease progression, where children’s capacity to articulate preferences was perceived as variable.

Respondents reported ethically sensitive situations in which parents encouraged participation despite a child’s apparent hesitancy, as well as cases in which children expressed interest in joining the study but parental concerns about burden or safety led to refusal of consent. These accounts did not necessarily indicate procedural failures but rather reflected the relational and emotionally charged contexts in which consent and assent decisions were made.

Interpreted in light of paediatric research ethics, these findings underscore the dynamic nature of assent and its close connection to emerging autonomy. Prior scholarship has emphasised that assent should not be understood as a one-time procedural step but as an ongoing process that is responsive to a child’s developmental stage, emotional state, and evolving preferences [7, 21, 22].

Several authors have argued that older children and adolescents may, depending on cognitive and emotional maturity, be capable of meaningful autonomous decision-making, warranting a more substantial role in consent processes [22]. The survey responses aligned with this ethical position in that HCPs consistently emphasised the importance of actively involving children in discussions about participation, even when legal consent authority rested with parents.

Within the FORTEe trial, respondents acknowledged the use of age-appropriate information materials, visual aids, and the explicit option to withdraw without consequences as important safeguards supporting children’s emerging autonomy.

### Perceived burden and the limits of acceptable participation

Importantly, respondents reported both burdens directly attributable to the trial and distress arising from the broader context of paediatric oncology care. While most respondents did not identify participation as substantially burdensome, the reported instances of questionnaire-related and logistical strain illustrate how research-related demands may be experienced alongside intensive clinical care.

### Fairness, randomisation, and access to potentially beneficial interventions

Random allocation to the control group emerged as a recurring source of ethical discomfort for both participants and HCPs. Respondents described situations in which children and families expressed disappointment or frustration when assigned to the control arm, particularly when exercise was perceived as beneficial or when intervention and control activities occurred in shared spaces. For some professionals, these situations generated moral unease, especially when caring for highly motivated children who were temporarily excluded from the intervention.

Such accounts reflect a well-recognised ethical tension in supportive care RCTs: although randomisation is methodologically justified, its experiential implications may be difficult to accommodate in vulnerable populations [12]. While the FORTEe trial employed established communication strategies and, where feasible, offered post-trial access to exercise programmes, respondents´ accounts suggest that these measures did not consistently prevent perceptions of unfairness.

Although respondents did not systematically propose specific alternative designs, their accounts of disappointment and perceived unfairness underscore the need for explicit communication and ethical reflection regarding randomisation. While no single design can be regarded as ethically optimal across all contexts, this reinforces the importance of clearly articulating and revisiting the ethical justification for randomisation during trial design, consent processes, and ongoing communication with families [23, 24]. From an ethical perspective, these accounts also point to more general tensions surrounding the use of randomised controlled designs in supportive care research involving highly vulnerable paediatric populations. Several respondents implicitly questioned the ethical acceptability of withholding an intervention perceived as beneficial, particularly when exercise was closely associated with wellbeing, coping, or normalcy during treatment. Such concerns align with broader ethical discussions about RCTs in contexts where equipoise may be difficult to sustain from the participant’s or clinician’s perspective, especially when interventions are experienced as low-risk and potentially beneficial rather than experimental [12, 25].

In this light, respondents’ accounts suggest the importance of considering how trial design choices and post-trial access arrangements are communicated and ethically justified. While alternative design approaches or enhanced post-trial access strategies may help mitigate perceptions of unfairness or moral distress, no single design can be regarded as ethically optimal across all settings. Our findings therefore highlight the need for explicit ethical reflection on these trade-offs during trial design and consent processes, particularly in supportive care research.

### Moral distress among HCPs and perceived distress among families

Moral distress was a recurring concern reported by respondents, particularly in relation to disease progression, relapse, or death. Many respondents described emotional strain when continuing trial-related interactions with children whose prognosis had worsened, or when communicating limitations of the intervention to families who had found exercise meaningful. These experiences align with established definitions of moral distress, which describe the psychological discomfort arising when professionals feel constrained from acting in ways they perceive as ethically appropriate [17].

At the same time, the findings suggest that not all distress reported by HCPs was attributed directly to trial participation. Professionals in paediatric oncology routinely encounter suffering and loss as part of their clinical roles, making it essential to distinguish between distress inherent to oncology care and distress that is intensified or shaped by research-specific decisions. This conceptual ambiguity reflects a well-recognised challenge in moral distress research, where the term is variably used to describe constraint-based ethical conflict as well as broader experiences of emotional and moral strain, particularly in high-burden clinical contexts [26–28].

Situations requiring trial-related judgments—such as discontinuation, non-re-enrolment, or withholding of an intervention—were frequently described as ethically challenging, highlighting the intersection of clinical care and research responsibilities [29].

In addition to their own experiences, respondents reported perceiving emotional and moral distress among patients and families, especially in contexts of relapse, bereavement, or unexpected deterioration. While these observations reflect HCPs’ interpretations rather than direct patient or parent reports, they nonetheless underscore the emotional and ethical complexity of conducting research alongside serious illness. Prior work has similarly highlighted the moral burden placed on families when serious paediatric illness coincides with high-stakes decision-making (e.g. parent moral distress) [30], and has also discussed related burdens in paediatric research and end-of-life contexts [31–33]. These findings reinforce calls for institutional structures that support ethical reflection, interdisciplinary communication, and psychosocial care—not only for families, but also for professionals navigating ethically demanding environments. Ethics debriefings, access to ethics consultation, and opportunities for reflective practice have been proposed as key mechanisms to mitigate moral distress and sustain ethical resilience among healthcare teams [34, 35].

### Ethical reflection beyond protocol adherence

Taken together, the findings demonstrate that ethical challenges in paediatric exercise-oncology trials extend beyond formal procedural requirements, such as informed consent or safety monitoring, and are shaped by relational dynamics, emotional labour, and structural constraints encountered in everyday practice. By foregrounding HCPs´ perspectives, this study illustrates how ethical considerations arise not only at the level of trial design and regulation, but also in ongoing interactions with children and families under conditions of serious illness.

These observations suggest that attention to ethical conduct in paediatric trials may require consideration of both procedural safeguards and the relational contexts in which research is embedded, particularly when interventions are delivered alongside intensive clinical care [8, 36–38].

### Strengths and limitations

This study has several limitations that warrant consideration. The achieved response rate of 45% (79 responses from 175 disseminated QR codes across various HCPs), is comparable to similar surveys among HCPs, but introduces the possibility of participation and selection bias. It is plausible that individuals with particularly strong views, whether favourable or critical, were more inclined to respond, potentially influencing the range of perspectives captured. Furthermore, the sampling frame, based on local trial staff lists, may have excluded professionals with more peripheral or informal involvement in the study. Nevertheless, the survey successfully reached a diverse cohort of HCPs from all ten FORTEe trial sites, enhancing the generalisability of findings across a variety of institutional contexts and healthcare systems.

The inclusion of both core FORTEe investigators and members of the broader clinical team, some of whom did not receive formal training on trial-specific procedures, may have resulted in variability in familiarity with the intervention and interpretation of survey items. While this heterogeneity may limit the comparability of some responses, it also represents a methodological strength. Including respondents with varying degrees of trial involvement provides a more ecologically valid account of the ethical challenges encountered in everyday practice and may have reduced social desirability bias by allowing more candid expressions of concern.

The survey was administered exclusively in English across all participating sites. Although this facilitated uniform distribution and avoided interpretive inconsistencies associated with multiple translations in a multinational study, it may have affected the clarity, nuance, or expressiveness of responses among non-native speakers.

The use of a web-based, time-efficient survey instrument inevitably limited the depth with which complex ethical reasoning could be explored. Compared to in-depth interviews or focus groups, the format may have constrained the expression of nuanced moral positions. However, the mixed-methods design, combining structured Likert-scale items with open-ended questions, enabled respondents to elaborate on salient ethical experiences while minimising response burden, which likely contributed to participation among busy clinical staff.

The cross-sectional design, implemented towards the end of the intervention period, limits insight into how ethical concerns may have evolved across different stages of the trial. At the same time, this timing ensured that most respondents had direct and contemporaneous experience with the intervention and its procedures, enhancing the immediacy and practical relevance of their reflections.

Importantly, several survey items used broad ethical terms, including *“moral distress”* and *“ethically relevant situations”*, without providing formal definitions within the questionnaire. As a result, responses may reflect variation in how participants interpreted these concepts, influenced by professional role, clinical experience, and linguistic background. Accordingly, findings related to moral distress should be understood as reflecting healthcare professionals’ self-reported perceptions of ethical and emotional strain rather than a uniform or clinically assessed construct. This limitation is particularly relevant when interpreting distress related to disease progression, which may overlap with the emotional demands of paediatric oncology care more generally. Similarly, reports of perceived burden and distress among patients and families must be interpreted with caution. Although respondents frequently distinguished between burdens directly attributable to trial participation and distress arising from the broader context of paediatric oncology care, the survey design does not allow for a clear causal attribution of distress to trial-related procedures. Consequently, some reported burden or distress may reflect the emotional and clinical realities of serious illness rather than effects uniquely associated with research participation.

Finally, although anonymity was guaranteed, the potential influence of social desirability cannot be entirely excluded. Respondents may have under-reported ethically problematic practices or overstated adherence to institutional policies. In addition, the study did not include the perspectives of patients or parents, limiting the findings to the professional domain. However, this limitation is partially addressed by recent complementary work within the FORTEe project that qualitatively explored the experiences of patients and families [39].

Despite these limitations, this study provides empirical insight into ethical challenges perceived by HCPs involved in a non-invasive behavioural intervention, an area that remains underrepresented in paediatric research ethics. By focusing on exercise oncology rather than pharmacological trials, the study broadens the scope of empirical ethics research and supports more context-sensitive approaches to the ethical conduct of paediatric trials.

## Conclusion

The FORTEe staff-survey indicates that HCPs perceived the ethical conduct of the trial predominantly positively. A large majority of respondents (86.1%) agreed or strongly agreed that the overall burden–benefit balance of trial participation was appropriate. However, the findings highlight several recurring ethical challenges encountered in practice, particularly in relation to the interpretation of child assent, parental influence in decision-making, perceptions of burden, fairness of randomisation, post-trial sustainability, and experiences of moral distress among healthcare professionals. Despite the implementation of procedural safeguards, including age-appropriate consent materials and the possibility of post-trial access to exercise support, respondents reported situations in which children and families experienced emotional strain, especially in the context of control-group allocation or disease progression. These observations underscore the importance of ongoing ethical attentiveness throughout trial implementation, rather than reliance on protocol-level safeguards alone.

Respondents also described moral distress among HCPs, particularly where trial-related decisions intersected with clinical deterioration or perceived unmet needs. While such distress must be interpreted in light of the broader emotional demands of paediatric oncology care, the findings suggest that structured opportunities for ethical reflection, communication support, and psychosocial resources may help professionals navigate ethically challenging situations more sustainably.

## Supplementary Information

Supplementary Material 1.

Supplementary Material 2.

Supplementary Material 3.

## Data Availability

Due to the inclusion of personal and sensitive information, the data underlying this study are not publicly available in accordance with data protection regulations. However, they may be obtained from the corresponding author upon reasonable request.

## Abbreviations

HCP: Health care professionals
RCT: Randomised controlled trial
