# Supplementary material for: Navigating ethical challenges in the FORTEe randomised controlled trial: a multi-centre staff survey on exercise intervention for children and adolescents undergoing cancer treatment
Source: BMC Med Ethics. 2026 Feb 25;27:45. doi: 10.1186/s12910-026-01414-6 (PMC12955331; doi:10.1186/s12910-026-01414-6)
Supplement: Supplementary file 1 — Supplementary Material 1. [file 12910_2026_1414_MOESM1_ESM.docx]

**Supplementary Material**

**Supplementary Table 1. Inclusion and exclusion criteria for participants in the FORTEe trial (as outlined in the Clinical Study Protocol, Version 1.4 May 2023) (16).**

| **Inclusion criteria** | **Exclusion criteria** |
| --- | --- |
| First/initial diagnosis of a **primary oncologic disease** according to the International Classification of Childhood Cancer (ICCC), requiring chemotherapy and/or radiation therapy **or**  First/initial diagnosis of a **relapsed oncologic disease** according to the International Classification of Childhood Cancer (ICCC), requiring chemotherapy and/or radiation therapy **or**  First/initial diagnosis of a **secondary oncologic disease** according to the International Classification of Childhood Cancer (ICCC), requiring chemotherapy and/or radiation therapy | Oncologic disease (primary/relapsed/secondary) according to the International Classification of Childhood Cancer (ICCC), requiring neither chemotherapy nor radiation therapy or solely requiring surgery |
| Age: **≥4 to ≤21 years** (completed 4 years of age to not completed 22 years of age) |  |
| Planned or started anti-cancer treatment (**chemo- and/or radiotherapy**) at one of the recruiting centres of the FORTEe trial | Reasons that already exist or are foreseeable at the time of study enrolment, due to which the patient will not have access to the exercise intervention and/or to the pre-test and/or to the post-test (e.g. foreseeable or planned, permanent change of treatment centre). |
| The patient is assessed by the treating team (paediatric oncologist, exercise professional etc.) as suitable to participate in the trial, e.g. due to medical or psychological reasons. | The patient is assessed by the treating team (paediatric oncologist, exercise professional etc.) as unsuitable to participate in the trial, e.g. due to medical or psychological reasons |
| Existing **informed consent (or assent)** to participate in the study   - For minor patients (≤ 18 years): The legal guardians assume that the child/adolescent is able to freely decide on participation or to consent to participation on the basis of the information received and have given written consent for participation in the study after detailed information and a time to consider (i.e. at least 24 hours). In addition to the informed consent given by the legal guardians, in adolescents ≥ 16 years of age, written consent to participate in the study is mandatory after detailed age appropriate information and after a time to consider. Documented assent is sought from all minor patients after age appropriate information. In the case of children > 6 years of age, this should, if possible, also be given as written informed consent. - For adult patients (≥18 years): Existing written consent to participate in the study after detailed information and after a time to consider (i.e. at least 24 hours). | After detailed information and, if necessary, having time to consider, the patient (≥ 16 years of age) did not agree to give written informed consent to participate in the trial.  In the case of minor patients (< 16 years of age): The legal guardians do not assume that the child/adolescent is able to freely decide on participation or to consent to participation on the basis of the information received, and do not consent to this themselves.  In case of minors capable of giving consent*: Lacking documented assent/ written informed consent.  ** in general minors are considered as capable of giving consent at the age of 14* |
| The patient (and the legal guardian) has/have sufficient knowledge of the respective national or English language so that it is possible to carry out the informed consent as well as interviews (in age-appropriate language). | The patient (and the legal guardians) has/have insufficient knowledge of the respective national or English language, so that it is not possible to carry out both the informed consent and interviews (in age-appropriate language). |
| The patient is not in a terminal phase of the disease.  *("Terminal" here refers to the last phase of the disease before dying, but does not automatically refer to patients who are defined and cared for as "palliative". Despite their assumed increased vulnerability, a general exclusion of palliative patients from the trial would provoke an inadequate bias of the study population and at the same time deny these patients a potentially enriching opportunity to participate.)* | The patient is in a terminal phase of the disease.  *("Terminal" here refers to the last phase of the disease before dying, but does not automatically refer to patients who are defined and cared for as "palliative". Despite their assumed increased vulnerability, a general exclusion of palliative patients from the trial would provoke an inadequate bias of the study population and at the same time deny these patients a potentially enriching opportunity to participate.)* |
