# Supplementary material for: Navigating ethical challenges in the FORTEe randomised controlled trial: a multi-centre staff survey on exercise intervention for children and adolescents undergoing cancer treatment
Source: BMC Med Ethics. 2026 Feb 25;27:45. doi: 10.1186/s12910-026-01414-6 (PMC12955331; doi:10.1186/s12910-026-01414-6)
Supplement: Supplementary file 2 — Supplementary Material 2. [file 12910_2026_1414_MOESM2_ESM.docx]

**Supplementary Material**

**Supplementary Information 2. English Version of the Questionnaire**

**Supplementary Survey**

| **Item** | **Content** | **Response Options** |
| --- | --- | --- |
| Profession | Please indicate your professional background | Physician / Oncologist  Nurse  Exercise professional  Psychologist  Social worker  Social scientist  Ethicist  Other |
| If you have another profession, please indicate: | *Free text* | — |
| Gender | Please indicate your gender | Male  Female  Other |
| Age Range | Please select your age group | 18–25 years  26–35 years  36–50 years  50+ years |
| Are you familiar with the FORTEe inclusion/exclusion criteria? | *Closed question* | Yes  No |
| Do you think the criteria for inclusion/exclusion will achieve the aims of the study? | *Closed question* | Yes  No |
| Please briefly describe why (if “no” to the previous question) | *Free text* | — |
| Are you familiar with the FORTEe informed consent process? | *Closed question* | Yes  No |
| Did you encounter any burden during the informed consent process? | *Closed question* | No  Yes, once  Yes, more than once  Not sure |
| Please describe why you (strongly) agree/disagree | *Free text* | — |
| Have you encountered ethically relevant situations regarding the relationship between patients and parents? | *Closed question* | No  Yes, once  Yes, more than once  Not sure |
| Please describe the situation(s) encountered | *Free text* | — |
| Did you witness ethically relevant events during exercise testing/training? | *Closed question* | No  Yes, once  Yes, more than once  Not sure |
| Please describe the situation(s) encountered | *Free text* | — |
| Did you witness any burdens on participants during questionnaires/interviews? | *Closed question* | No  Yes, once  Yes, more than once  Not sure |
| Please describe the situation(s) encountered | *Free text* | — |
| The balance of burden and benefit of participation was appropriate for participants | *Likert scale* | Strongly agree  Agree  Disagree  Strongly disagree  Not sure |
| Please describe why you (strongly) agree | *Free text* | — |
| Please describe why you (strongly) disagree | *Free text* | — |
| Participants’ needs for exercise are adequately met after completing the trial | *Likert scale* | Strongly agree  Agree  Disagree  Strongly disagree  Not sure |
| Please describe why you (strongly) agree | *Free text* | — |
| Please describe why you (strongly) disagree | *Free text* | — |
| Did you encounter difficulties explaining the lack of exercise sessions post-trial? | *Closed question* | Yes, frequently  Yes, a few times  Yes, once  No, because our patients had access  No, no difficulties |
| Please describe the situation(s) encountered | *Free text* | — |
| Have you encountered difficult situations managing the control group? | *Closed question* | No  Yes, once  Yes, more than once  Not sure |
| Please describe the situation(s) encountered | *Free text* | — |
| Did any patients encounter cancer progression, relapse, or death during participation? | *Closed question* | No  Yes, once  Yes, more than once  Not sure |
| Did this situation cause moral distress? | *Closed question* | Yes, among the staff  Yes, to the patients/family  No |
| Moral distress among staff regarding the trial | *Closed question* | Appropriateness / Additional burden  Discussing participation  Communication  Other |
| Please describe the situation that caused moral distress among staff | *Free text* | — |
| Moral distress among patients/family regarding the trial | *Closed question* | Appropriateness / Additional burden  Discussing participation  Communication  Other |
| Please describe the situation that caused moral distress among patients/family | *Free text* | — |

*This document presents the complete English version of the questionnaire used in the study.
The structure and wording correspond to the final survey instrument administered to participants.
Question types (e.g. multiple choice, Likert scale, or free text) are indicated for clarity.*
