# Supplementary material for: Navigating ethical challenges in the FORTEe randomised controlled trial: a multi-centre staff survey on exercise intervention for children and adolescents undergoing cancer treatment
Source: BMC Med Ethics. 2026 Feb 25;27:45. doi: 10.1186/s12910-026-01414-6 (PMC12955331; doi:10.1186/s12910-026-01414-6)
Supplement: Supplementary file 3 — Supplementary Material 3. [file 12910_2026_1414_MOESM3_ESM.docx]

# Supplementary Material

**Supplementary Table 3. Survey domains, conceptual focus, and corresponding survey items**

| **Survey domains** | **Conceptual focus** | **Closed-ended items** | **Open-text items** |
| --- | --- | --- | --- |
| Inclusion and exclusion criteria | HCP familiarity with and ethical appraisal of eligibility criteria | Familiarity with criteria; perceived ethical appropriateness | Comments on clarity or concerns |
| Informed consent and assent | Experiences with consent processes and perceived burden | Familiarity with consent process; perceived burden | Ethical concerns related to consent |
| Parent–child decision-making dynamics | Ethically relevant tensions between parents and children | Encountered ethically relevant situations | Description of parent–child dynamics |
| Ethical issues during exercise testing and training | Safety, assent, and ethically sensitive situations during intervention delivery | Observed ethically relevant events | Description of specific situations |
| Perceived burden–benefit balance | Burden associated with questionnaires, testing, and participation | Perceived burden; burden–benefit balance | Description of burden experiences |
| Sustainability and post-trial access | Continuity of exercise provision after trial completion | Adequacy of post-trial exercise provision | Barriers and facilitators of sustainability |
| Fairness of group allocation | Ethical challenges related to randomisation and control group assignment | Difficulties explaining group allocation | Description of fairness-related concerns |
| Moral distress among HCPs | Experiences of moral distress related to trial participation | Encountered moral distress; frequency | Description of morally distressing situations |
| Perceived distress among patients and families (HCP perspective) | HCP perceptions of distress in patients or families | Observed distress | Description of perceived distress |

*This table summarises the predefined survey domains used to structure data collection and analysis, their conceptual focus, and the corresponding closed-ended and open-text survey items. Domains served as descriptive analytic groupings rather than normative ethical frameworks and informed both quantitative reporting and the organisation of qualitative free-text responses.*
